# New Potential Weapons for Refractory Scleritis in the Era of Targeted Therapy

**DOI:** 10.1155/2020/8294560

**Published:** 2020-01-17

**Authors:** Claudia Fabiani, Jurgen Sota, Maite Sainz-de-la-Maza, Laura Pelegrín, Giacomo Emmi, Giuseppe Lopalco, Florenzo Iannone, Lorenzo Vannozzi, Silvana Guerriero, Maria Chiara Gelmi, Donato Rigante, Gian Marco Tosi, José Hernández-Rodríguez, Luca Cantarini

**Affiliations:** ^1^Ophthalmology Unit, Department of Medicine, Surgery and Neuroscience, University of Siena, Siena, Italy; ^2^Research Center of Systemic Autoinflammatory Diseases and Behçet's Disease, Rheumatology Unit of the Department of Medical Sciences, Surgery and Neurosciences, University of Siena, Siena, Italy; ^3^Clinical Institute of Ophthalmology, Hospital Clinic of Barcelona, IDIBAPS, University of Barcelona, Barcelona, Spain; ^4^Department of Experimental and Clinical Medicine, University of Florence, Florence, Italy; ^5^Rheumatology Unit, Department of Emergency and Organ Transplantation (DETO), University of Bari, Bari, Italy; ^6^Department of Surgery and Translational Medicine, Eye Clinic, University of Florence, Florence, Italy; ^7^Department of Ophthalmology and Otolaryngology, University of Bari, Bari, Italy; ^8^Institute of Pediatrics, Università Cattolica Sacro Cuore, Fondazione Policlinico Universitario A. Gemelli I.R.C.C.S., Rome, Italy; ^9^Vasculitis Research Unit and Autoinflammatory Diseases Clinical Unit, Department of Autoimmune Diseases, Hospital Clinic of Barcelona, IDIBAPS, University of Barcelona, Barcelona, Spain

## Abstract

**Objective:**

To assess the efficacy of biologic drugs, beyond tumor necrosis factor- (TNF-) *α* inhibitors, in the management of noninfectious refractory scleritis, either idiopathic or associated with systemic immune-mediated disorders. *Patients and Methods*. This is a retrospective study assessing the efficacy of several biologic agents (rituximab, anakinra, tocilizumab, and abatacept) and the small molecule tofacitinib in the treatment of scleritis through assessment of scleral inflammation and relapses, as well as treatment impact on best-corrected visual acuity (BCVA) and safety profile.

**Results:**

Fourteen patients (19 eyes) were enrolled in the study. Scleritis inflammatory grading significantly improved from baseline to 3 months (*p* = 0.002) and from baseline to the last follow-up visit (*p* = 0.002). Scleritis relapses significantly decreased between the 12 months preceding and following biologic therapy (*p* = 0.007). No differences regarding BCVA were observed (*p* = 0.67). Regarding adverse events, only one patient developed pneumonia and septic shock under rituximab treatment.

**Conclusions:**

Our results, though limited to a low number of patients, highlight the effectiveness of different biologic therapies in the treatment of noninfectious refractory scleritis, showing to control scleral inflammation and allowing a significant reduction in the number of relapses.

## 1. Introduction

Noninfectious scleritis is a severe inflammatory disease of the white outer coating of the eye frequently associated with underlying systemic inflammatory diseases, such as rheumatoid arthritis, systemic lupus erythematosus, relapsing polychondritis, and systemic vasculitides [1, 2]. The most aggressive forms of scleritis, such as necrotizing scleritis and posterior scleritis, represent conditions at high risk of serious functional and anatomical sequelae. The most dreaded complication of scleritis is perforation, which can lead to loss of the eye [1]. Moreover, damage to contiguous inflamed ocular structures such as cornea, uvea, and retina may also occur and leave permanent scarring responsible for irreversible visual impairment. Early diagnosis in these cases is paramount, as aggressive treatment with systemic high-dose glucocorticoids (GCs) in the acute phase and long-term conventional disease-modifying antirheumatic drugs (cDMARDs) on the long term is required [1]. In refractory and most severe cases, several biologics have been employed to control scleral inflammation. Among biologic agents, tumor necrosis factor- (TNF-) *α* inhibitors have shown to induce a complete and rapid control of scleral inflammation within a few weeks from the start of treatment [3, 4]. Beyond TNF-*α* inhibition, a prospective randomized double-masked trial by Suhler et al. found that the anti-CD 20 monoclonal antibody rituximab is effective and well tolerated during a 24-week follow-up period [5]. However, only small case series or isolated case reports have been reported on the use of other different biologics [6–11].

In this regard, we report herein our experience on the effectiveness of several different biologic agents, with mechanism of action different from TNF-*α* inhibitors, in the management of noninfectious recalcitrant scleritis.

## 2. Patients and Methods

### 2.1. Study Participants and Screening Methodology

We conducted a retrospective evaluation of patients attending four tertiary ophthalmologic and rheumatologic clinics for the management and treatment of inflammatory ocular and systemic diseases who were affected by noninfectious scleritis and treated with biologic agents with mechanism of action different from TNF-*α* inhibitors. Patients with scleritis effectively treated with systemic TNF-*α* inhibitors were not included in this study. Treatment with biologics was established for both active noninfectious refractory scleritis and/or uncontrolled systemic disease associated with scleritis.

The study was approved by the Local Ethic Committee (Prot. N 14951) and adhered to the tenets of the Declaration of Helsinki. A written informed consent was obtained by all study participants or their legal guardians. Patients were screened for latent or active infections before starting the biologic agent with exams including chest radiography, Mantoux or QuantiFERON tests, HBV, HCV, HIV, syphilis, Borrelia burgdorferi serologies, and urine culture.

The following demographic, clinical, and therapeutic data were retrospectively collected: age, sex, class I human leukocyte antigen, age at scleritis onset, disease duration, scleritis relapses, ocular complications, preceding biologic therapy and cDMARDs, preceding local or systemic GCs, and adverse events (AEs). Patients were regularly examined every 3 months and in case of necessity (AEs or disease flare) by either the ophthalmologist or the rheumatologist/internist.

Our study is aimed at evaluating the efficacy of different biologic agents, beyond TNF-*α* inhibition, in terms of control of scleral inflammation, number of ocular relapses, GC-sparing effect, and visual acuity. Moreover, we recorded the safety profile of therapies and assessed any ocular complication occurring during treatment.

### 2.2. Ophthalmologic and Systemic Work-Up

All study participants underwent regular complete ophthalmologic examinations and systemic work-up assessments. Ophthalmologic examination included evaluation of best-corrected visual acuity (BCVA), measurement of intraocular pressure, complete slit lamp examination, and fundus examination. Optical coherence tomography was performed to establish any morphologic macular change at a retinal and choroidal level. Ocular ultrasonography and/or orbit MR scan were performed to confirm the diagnosis of posterior scleritis. Anatomical pattern of scleritis was classified according to the scheme proposed by Watson and Hayreh [12], whereas scleral inflammation was evaluated according to the scleritis grading system proposed by Sen et al. [13], with a score ranging from 0 to 4+. An extensive multidisciplinary work-up was also performed to investigate for a potential underling systemic disease.

### 2.3. Statistics

Data were analyzed using IBMSPSS Statistics for Windows, version 24 (IBM Corp., Armonk, NY, United States). Descriptive statistics was employed to display mean and standard deviation (SD) or median and interquartile range (IQR) as appropriate. Normality was assessed by Shapiro–Wilk test. Repeated ordinal data were computed with Friedman test followed by post hoc Wilcoxon rank sum test. Means were compared by unpaired *t*-test or Mann-Whitney *U* test as needed. The threshold for statistical significance was set to *p* < 0.05, and all *p* values were two sided.

## 3. Results

Nineteen eyes of 14 patients were included in the study. Most patients were from Caucasian ancestry (85.7%) except 2 (1 Afro-American and 1 Hispanic). Female patients represented 57% of the study participants. Demographic data, type of eye involvement, and associated systemic diseases are summarized in Table 1 whereas a detailed description of clinical characteristics and treatment data of the 14 patients enrolled is provided in Tables 2 and 3. Biologic therapy was initiated for active scleritis in 9 out of 14 patients (64.3%) and the remaining 5 because of the associated systemic disease (rheumatoid arthritis in 4 patients and granulomatosis with polyangiitis in one patient). Median ± IQR treatment duration was 13.5 ± 25.50 months, with a minimum of 1 month, a maximum of 56 months, and a range of 55 months.

The scleritis grade significantly decreased during the follow-up period (*p* < 0.0001). More in detail, a significant decrease was detected between baseline and 3 months (median ± IQR2 ± 4 and 0 ± 1, respectively, *p* = 0.002) and between baseline and the last follow-up visit (median ± IQR2 ± 4 and 0 ± 1, respectively, *p* = 0.002), while no significant differences emerged between 3 months and the last follow-up visit (*p* = 0.414). Resolution of scleritis was achieved in 10 out of 19 eyes. In the remaining 9 eyes, scleritis improved in 2 eyes, remained quiescent in 4 eyes, did not improve in 2 eyes, and worsened in 1 eye.

A significant decrease in the number of scleritis relapses between the 12 months preceding and following biologic therapy was identified (*p* = 0.007). A GC-sparing effect was also observed (mean GCs before treatment was 15.13 ± 9.25 mg/daily of prednisone or equivalent; mean GCs at last follow-up was 5.14 ± 4.63 mg/daily of prednisone or equivalent; *p* = 0.016).

With regard to AEs, only one patient developed a serious adverse event (pneumonia and septic shock) 1 month after the introduction of rituximab. The following ocular complications developed in 6 eyes: cataract (*n* = 3), scleral thinning (*n* = 3), infectious keratitis that required tectonic patch because of impending perforation (*n* = 1), and macular subatrophy (*n* = 1). No significant differences regarding BCVA values were observed between baseline and the last follow-up visit (median ± IQR10 ± 3 and 10 ± 1, respectively) (*p* = 0.67).

## 4. Discussion

The advent of biologic therapy has revolutionized the management of noninfectious intraocular inflammation. However, most of the reported literature is primarily focused in the treatment of uveitis [14], whereas the efficacy and safety of biologics in scleritis have mainly been addressed by single case reports and small case series [6–11, 15].

Based on our findings, therapy with different biologic agents has resulted in a rapid control of scleral inflammation within 3 months from the start of treatment. Similar results were reported in other studies as well. Treatment with the interleukin- (IL-) 1 receptor antagonist anakinra resulted in a rapid improvement within 1 month in a case series of 10 consecutive patients affected by severe and refractory nonnecrotizing scleritis [7]. Silpa-Archa et al. reported the achievement of inflammation control with steroid-sparing effect in 50% of their scleritis patients under the IL-6 inhibitor tocilizumab. They also observed a faster response to tocilizumab in scleritis compared to patients with uveitis [10].

In our cohort, alongside the rapid efficacy in controlling scleral inflammation, scleritis grade decreased significantly also from baseline to the last follow-up visit, which advocates for a prolonged effectiveness over time. Additionally, we also observed a significant decrease in the number of scleritis relapses.

Among biologic treatment, B cell-depletion therapy seemed to be another feasible option in achieving a longstanding scleritis remission [5, 11]. Indeed, Suhler et al. found the anti-CD 20 monoclonal antibody rituximab to be effective and well tolerated on a 24-week period in patients affected by refractory scleritis. Interestingly, no notable differences in terms of efficacy and toxicity were found between patients receiving 500 mg and those receiving 1000 mg of rituximab [5]. In a retrospective case series of 15 patients with a mean follow-up of 34 months, rituximab was shown to be effective for recalcitrant noninfectious scleritis and in some cases resulted in a long-term durable drug-free remission. However, the authors stated that aggressive rituximab regimens with higher dosages were required to maintain steroid-free remission [11]. One of our patients (patient 4) treated with rituximab did not show any improvement of scleral inflammation. She was initially diagnosed with an orbital inflammatory disease and was therefore prescribed rituximab based on recent reports of its efficacy in this condition [16, 17]. She is now under investigation to start an anti-TNF-*α* monoclonal antibody.

In this regard, we could not evaluate any potential statistical difference between various treatment regimens, due to the low number of patients treated with rituximab included in our study.

Concerning to visual function, 14 out of 19 eyes (73.68%) presented with a BCVA ≥ 9 at baseline. Therefore, the lack of significant differences between baseline and the last follow-up visit suggests an ability of biologic therapy to preserve visual acuity over time in scleritis patients.

To the best of our knowledge, we report the first cases of refractory scleritis treated with the recombinant fusion protein abatacept and the Janus kinase inhibitor tofacitinib. More in detail, among the 4 patients (6 eyes) treated with either abatacept or the small molecule tofacitinib, all had an active anterior diffuse scleritis at baseline and all but one achieved resolution of ocular inflammation at the last follow-up visit.

Several biologic agents with a different mechanism of action from anti-TNF-*α* inhibitors were found to be effective in most of our patients. However, in some cases, the abovementioned drugs did not show the same efficacy. This is likely to be explained by the heterogeneity of our sample determining distinct pathogenetic backgrounds in accordance to different systemic diseases encountered in our cohort. Therefore, in addition to its anatomical pattern, scleritis treatment should be ideally tailored also by taking into account the associated systemic disorder. In case of idiopathic scleritis, the response to treatment may vary due to the possible activation of different unknown underlying pathogenetic pathways.

However, with the expanding number of biologics available, the prognosis of scleritis, either idiopathic or associated with systemic immune-mediated disorders, may experience a radical change.

To date, we are far from drawing firm conclusions, and our current limited knowledge warrants further studies prospectively designed with larger samples of patients to shed light on this topic. Indeed, our present study comprises several limitations, including its retrospective design, the small sample size, and therapeutic heterogeneity with different biologic agents employed before study entry and within study period.

In conclusion, our results highlight the effectiveness of several biologic agents in the treatment of noninfectious refractory scleritis, showing their potential ability in controlling scleral inflammation and determining a significant reduction in the number of relapses, while preserving visual acuity and displaying an excellent safety profile.

## Figures and Tables

**Table 1:** Demographic and clinical features of our cohort of 14 patients.

Demographic, laboratory, and clinical data	Mean ± SD
Age (years)	47.29 ± 17.73
Age at scleritis onset (years)	42.57 ± 18.54
Disease duration (years)	5.00 ± 3.01
HLA (*N*)	HLA-B51 (4)
	HLA-B27 (1)
	HLA-B35 (1)
Female/male (*N*)	8/6
Eye disease	*N* eyes
Anterior diffuse scleritis	11 (57.89%)
Anterior nodular scleritis	4 (21.05%)
Anterior necrotizing scleritis	1 (5.26%)
Posterior scleritis	3 (15.80%)
Concomitant uveitis	4 (4 AU)
Associated keratitis	3 PUK
Associated systemic disease	
Rheumatoid arthritis	7
Granulomatosis with polyangiitis	2
Microscopic polyangiitis	1
Psoriatic arthritis	1
Familial Mediterranean fever	1

AU: anterior uveitis; HLA: human leukocyte antigen; PUK: peripheral ulcerative keratitis; SD: standard deviation.

**Table 2:** Clinical characteristics and past treatments of 14 patients enrolled.

Patient	Age/sex	Anatomical pattern	Laterality	Systemic disease	Preceding biologics and their dose
1	42/M	Nodular AS	Bilateral	GPA	—
2	76/M	Necrotizing AS	Unilateral	MPA	—
3	66/M	Diffuse AS	Unilateral	RA	—
4	16/F	Posterior scleritis	Unilateral	Idiopathic	—
5	32/F	Diffuse AS	Unilateral	GPA	—
6	56/F	Diffuse AS	Bilateral	RA	ADA (40 mg/2 weeks), ABA (125 mg/week), IFX (5 mg/kg/4 weeks)
7	46/M	Diffuse AS	Unilateral	PsA	ADA 40 mg/2 weeks, IFX (5 mg/kg/4 weeks)
8	18/F	Posterior scleritis	Unilateral	Idiopathic	RTX (2 gr/6 months)
9	59/M	Nodular AS	Bilateral	RA	—
10	66/M	Posterior scleritis	Unilateral	FMF	—
11	54/F	Diffuse AS	Bilateral	RA	ADA (40 mg/2 weeks)
12	52/F	Diffuse AS	Unilateral	RA	—
13	34/F	Diffuse AS	Unilateral	RA	—
14	45/F	Diffuse AS	Bilateral	RA	ETN (50 mg/week), ADA (40 mg/2 weeks), TCZ (162 mg/week), RTX (2 gr/6 months)

ABA: abatacept; ADA: adalimumab; AS: anterior scleritis; F: female; GPA: granulomatosis with polyangiitis; IFX: infliximab; M: male; MPA: microscopic polyangiitis; PsA: psoriatic arthritis; RA: rheumatoid arthritis; RTX: rituximab; TCZ: tocilizumab.

**Table 3:** Current treatment and ocular data regarding scleritis grading and number of relapses in our cohort of patients.

Patient	Ongoing treatment and dosage	Indication	Treatment duration (months)	Scleritis grading before therapy RE	Scleritis grading at last follow-up RE	Scleritis grading before therapy LE	Scleritis grading at last follow-up LE	No. of relapses 12 months preceding therapy	No. of relapses after 12 months of therapy	No. of relapses at last follow-up	cDMARDs	GCs^§^	AE
1	RTX^∗^	Scleritis	7	2	0	0	0	3	NC	0	MFA	10	—
2	RTX 2 gr	Scleritis	1	—	—	4	0	3	NC	0	—	—	^#^
3	RTX^∗^	Scleritis	15	—	—	4	0	3	0	—	LFN	5	—
4	RTX 2 gr/6 months	Scleritis	12	3	4	—	—	3	—	—	—	—	—
5	RTX 2 gr/6 months	Systemic	30	2	0	—	—	1	0	—	AZA	—	—
6	TCZ 162 mg/week	Scleritis	3	0	0	2	0	3	NC	0	—	—	—
7	TCZ 162 mg/week	Scleritis	28	2	0	—	—	4	1	—	—	5	—
8	ANA 100 mg/day	Scleritis	1	4	3	—	—	3	NC	0	—	2.5	—
9	ANA 100 mg/day	Scleritis	3	4	0	0	0	3	NC	0	MTX	12.5	—
10	ANA 100 mg/day	Scleritis	44	—	—	1	0	1	0	—	—	—	—
11	ABA 125 mg/week	Systemic	24	4	4	3	3	4	3	—	—	—	—
12	ABA 125 mg/week	Systemic	56	—	—	1	0	1	0	—	MTX	—	—
13	ABA 125 mg/week	Systemic	15	—	—	2	0	1	0	—	MTX	—	—
14	TFC 5 mg twice daily	Systemic	6	0	0	2	0	1	NC	—	—	5	—

ABA: abatacept; ANA: anakinra; AE: adverse event; AZA: azathioprine; cDMARDs: conventional disease-modifying antirheumatic drugs; GCs: glucocorticoids; LE: left eye; LFN: leflunomide; MFA: mycophenolic acid; MTX: methotrexate; No.: number; NC: not calculable; RE: right eye; RTX: rituximab; TCZ: tocilizumab; TFC: tofacitinib. ^∗^2 loading dose infusions of 1000 mg separated by 2 weeks and 500 mg every 6 months thereafter. ^#^Pneumonia and septic shock. ^§^Daily intake of glucocorticoids (prednisone or equivalent expressed in mg).

## Data Availability

The data used to support the findings of this study are available from the corresponding authors upon request.
